# Creating Rat Model for Hypoxic Brain Damage in Neonates by Oxygen Deprivation

**DOI:** 10.1371/journal.pone.0083589

**Published:** 2013-12-17

**Authors:** Qiaoli Zhang, Yingxue Ding, Yanqing Yao, Yang Yu, Lijun Yang, Hong Cui

**Affiliations:** Department of Pediatrics of Beijing Friendship Hospital, Capital Medical University, Beijing, P. R. China; Universidade de São Paulo, Brazil

## Abstract

Current study explores the feasibility of using a non-surgical method of oxygen deprivation to create Hypoxic brain damage in neonatal rats for medical studies. 7-day-old Sprague Dowley (SD) rats were kept in a container with low oxygen level (8%) for 1.5h. A second group had bilateral cephalic artery ligation before the 1.5h-low oxygen treatment, a method similar to the popular Rice method, to expose the brain to both hypoxic and ischemic situations. Short term neural functions and brain water weights were evaluated 1 day after the hypoxic treatment. Brain pathology and histology were also examined at 1 day and 3 days after the hypoxic treatment. Both groups showed impaired neural functions and increased brain water weight compared to the controls. Histology studies also revealed injuries in the subcortex, hippocampus and lateral ventricle in the brains from both groups. There is no significant difference in the degree of brain damages observed in the two groups. Our work demonstrated that oxygen deprivation alone is sufficient to cause brain damages similar to those seen in Hypoxic-ischemic brain disease (HIBD). Because this method avoids the invasive surgical procedure and therefore reduces the stress and mortality of laboratory animals during the experiment, we recommend it to be the favorable method for creating rat models for HIBD studies.

## Introduction

Hypoxic-ischemic brain disease (HIBD) is the brain damage in newborn infants caused by partial or total hypoxia and decreased cerebral blood supply due to perinatal asphyxia [1]. Although full recovery can be expected in mild cases, death or serious sequelae in the nervous system [2,3] may happen in severe cases. The disease has an incidence rate of 1/ 1000 to 2/ 1000 in full term infants [4], and the mortality is 15 % to 20 %. About 25 % to 30 % of the survivors have permanent neural defect such as cerebral palsy, epilepsia, memory deficiency, hypophrenia, etc [5-7]. No standard, treatment method has been developed and the pathogenesis of the condition has not been completely understood. 

Research on HIBD is difficult due to the lack of an effective animal model for the disease. The Rice method, which was first created by Rice et al [8] in 1981, is currently the most commonly used method for the preparation of HIBD animal model. The procedure requires unilateral ligation of carotid artery in a 7d old rat followed by 2.5 to 3h of low oxygen concentration (8%) treatment. This method does simulate the newborn HIBD. However, it has several disadvantages: first, the bilateral internal carotid system of SD rat is joined together at the basilar artery. Therefore, unilateral ligation of carotid artery is not likely to initiate pathological changes effectively. Second, the unilateral ligation surgery is invasive and may cause partial ischemic injury in the brain during the operation. Third, it is a difficult procedure and trauma caused by the surgery and the anesthesia procedure is tremendous for neonatal rats. Moreover, it does not effectively duplicate the occurrence and the development process of HIBD in humans. Thus finding a simple and convenient method for creating neonatal rat model for advanced studies newborn HIBD is critical and imperative. Congle Zhou [9] et al have successfully created porcine models for brain anoxia by oxygen deprivation alone as well as by combining bilateral carotid artery ligation and oxygen deprivation. They have demonstrated that various degrees of cerebral injury can be observed 72h after anoxia and there was no significant difference in the physiological parameters, cerebral blood flow and cerebral perfusion in newborn pigs after the two procedures. This shows that oxygen deprivation alone is sufficient to simulate HIBD conditions in newborn pigs and surgical ligation of carotid arteries may not be necessary. 

Presently rats are still favored as animal model for HIBD studies over pigs because of their smaller size, shorter reproductive cycle and lower maintenance cost. They also are a better model for long-term neuropathologic and behavioral studies. Nonetheless, creating rat HIBD model without carotid artery ligation has not been attempted. In this study we compared effectiveness of replicating HIBD symptoms by oxygen deprivation alone vs. combining oxygen deprivation with bilateral carotid artery ligation. The results will be used to evaluate the feasibility of preparing neonatal rat HIBD model by a simple, non-invasive oxygen deprivation method.

## Material and Methods

All animal procedures have been approved by the Ethics Committee of Capital Medical University. Anesthesia and surgical protocols were all optimized to minimize handling of the animals and reduce trauma.

### Animals

The minimum number of animals needed for the study was determined by power analysis and a total of 120 Sprague Dowley rats aged 7 days (10 to 12g in bodyweight) were obtained from Beijing Vital River Experimental Animal Techology Co.,Ltd. They were evenly divided in to three groups by random selection: the controls, the hypoxic group, and the ischemic+hypoxic group.

Induction of hypoxic brain injuries: rats from the hypoxic group were placed in a closed container. The container was then incubated in a 37°C water bath and aerated with air containing 8% O2 and 92 % N2 for 1.5h. 

The ischemic+hypoxic group underwent bilateral cephalic artery ligation after ether anesthesia. Before the surgery, animals were anesthetized by intra-peritoneal injection of 4% chloral hydrate (0.5ml/100g bodyweight). A 0.5 cm incision was made at the ventral side of the neck, through which the cephalic arteries were isolated and ligated. The incision was then closed with stitches. Afterwards, the animals were placed in an incubation chamber (37°C) for 10 min until their body temperature and behavior returned turned to normal. They were allowed to rest for another hour before being placed in the container aerated with 8% O2 and 92 % N2 for 1.5h at 37°C. The control group received no surgery or low oxygen treatment.

### Neural function evaluation

Two behavior tests, the righting reflex and the geotactic reflex, were performed on 8 randomly selected rats from 1d after the treatment. In the righting reflex test, rats were placed on their back and the time it took for them to flip to the prone position with their paws on the floor was recorded. In the geotactic reflex test: the rats were placed on the 45° slope made of a wood panel 30 cm in length and covered with a wet towel. The rats were positioned with their heads facing downslope at the center of the wood panel. The time it took for them to turn around and face upslope (torsion angle > 90°) were recorded [10].

Two long term behavior tests, the vertical screen test and the grid walk test, were performed 21d after the treatment. In the vertical screen test, the rats were placed on a mesh wire screen (55.2x22.5cm). The screen was then rotated from horizontal position to vertical position in less than 2s. The time each rat remained clinging on the screen were recorded. In the grid walk test, rats were required to walk on awire mesh grid (3x3cm grid squares, 29x29 total area) for 2min. One foot miss was counted when the entire claw stuck through the grid. Test scores were calculated for both front and hind limbs as the ratio of misses over total steps taken [11]. 

### The determination of brain water content

1d after the experiment treatment, 8 rats were randomly selected from each group and euthanized by decollation. Brains were collected immediately by craniotomy. Wet weights of the brains were recorded using an electronic balance (AR1140, OHAUS, USA) within 2min of the dissection under room temperature. The brains were dried at 98°C in an oven for 48h and the dry weights were measured after air cooling. The brain water contents were calculated by the Elliot formula: brain water content (%) = (brain wet weight - brain dry weight)∕brain wet weight×100%.

### Serum CK and LDH level

Blood were collected from the rats sacrificed 1d after the treatment. The activities of CK and LDH in the serum were quantified using an Architect I-2000 immunology analyzer.

### Expression of inflammatory mediators TNF-α, IL-1B and MMP-9 in the brain

Another set of 8 rats were sacrificed by decollation 1d after the treatment and their brains were saved. Brain tissues were homologized in (buffer) at 1:9 w/v ratio and TNF-α, IL-1B and MMP-9 concentration in the homogenates were determined by commercial ELISA kits (R & D Systems, Minneapolis, MN, USA). 

### Histology studies

8 of the remaining rats were sacrificed at 1d, 3d and 21d after the treatment. Brains were removed and immediately fixed in neutral buffered formalin for several days. The fixed brains were paraffin embedded and sectioned after dehydration and vitrification. The slides were stained with hematoxylin fluid for 10min after xylene de-wax and graded ethanol debenzolization. They were then washed with tap water, differentiated with hydrochloric acid and ethanol before staining with eosin for 2min. After graded ethanol dehydration and xylene vitrification, the slides were sealed and ready for examination.

### Pathological quantification of HE stained brain tissues

Slides from the cortex, hippocampus and lateral ventricle were observed under 400X microscope. For each brain part, 5 areas were randomly selected for image analysis. Pathological conditions were graded with Foster’s pathological standard designed for neonatal pigs and the final score of is the sum of the scores of the individual areas.

### *In situ* apoptosis analysis

Cell death in the brain 1d after the treatment was analyzed using the fluorescein *In Situ* Cell Death Detection Kit from Roche (Mannheim, Germany). Slides from the brains were dewaxed and rehydrated before incubating in proteinase K at 37°C for 30 min. After washing twice with PBS, the slides were labeled following the manufacturer’s protocols and observed under a fluorescence microscope.

### Expression of c-fos and p-ERK in the brain

The expression of c-fox and p-ERK in the brain 1d after treatment were analyzed using immunohistochemistry. The slides from the brains were dwaxed and rehydrated. They were first incubated with 1:100 rabbit anti c-fos or p-ERK primary antibodies (Santa Cruz Biotechnology, Dallas, TX, USA) overnight at 4°C and then 1:100 enzyme conjugated secondary antibody (Santa Cruz Biotechnology, Dallas, TX, USA) for 30min at 37 °C. After DAB color development, the slides were observed under the microscope.

### Statistical analysis

Data were analyzed by multi-factor analysis of variance (ANOVA) followed by post hoc Bonferroni test by SPSS13.0. P-values < 0.05 were considered statistical significant.

## Results

### Prolonged reflex time of hypoxic groups in neural function evaluation

1d after the surgery and low oxygen treatment, both the hypoxic group and the hypoxic+ischemic group recorded significantly longer reflex time for the righting reflex test and the geotactic reflex test compared to the controls while there is no significant difference between the two groups in either of the tests (Table 1).

**Table 1:** Short-term neural function determination (N=8, in each group).

groups	time needing to finish righting reflex (s)	time needing to finish geotactic reflex (s)
The controls	2.13±0.64	6.25±0.89
The hypoxic group	5.50±0.93^**^	16.13±0.83^**^
The ischemic+hypoxic group	5.75±0.89^**^	16.75±1.04^**^

^**^, p<0.01 VS the controls

At 21d after the treatment, rats in the hypoxic group and hypoxic+ischemic group both were significantly less efficient in the vertical screen test and the grid walk test compared to the controls. Rats recorded much shorter time in the vertical screen test and more frequent missed foot in the grid walk test. However, no significant differences were observed between the hypoxic and the hypoxi+ischemic group in either test (Table 2). 

**Table 2:** Vertical screen test and the grid walk test result in each group.

	Vertical screen test	Grid walk test	
groups	Time of seizing screen (s)	Forelimb step error rate (%)	Hind step error rate (%)
The controls	27.8±0.4	1.2±0.2	0.9±0.1
The hypoxic group	4.8±0.7^**^	2.6±0.3^**^	2.2±0.3^**^
The ischemic+hypoxic group	4.6±0.6^**^	2.8±0.2^**^	2.2±0.2^**^

^**^ p<0.01 VS controls

### Higher brain water content in hypoxic and ischemic+hypoxic group

There is no significant difference in the brain water content between the hypoxic group and the ischemic+hypoxic group but both were significantly higher than the controls (Table 3).

**Table 3:** Determination of brain moisture content of neonate rat (N=8, in each group).

groups	Brain moisture content（%）
The controls	87.46±0.33
The hypoxic group	88.59±0.43^**^
The ischemic+hypoxic group	88.61±0.41^**^

^**^ p<0.01, VS the controls.

### Elevated serum CK and LDH level in hypoxic and ischemic+hypoxic group

Compared to the controls, serum CK level was significantly elevated in both hypoxic and ischemic+hypoxic group (p<0.01). Similary, serum LDH level also was significantly elevated in the two treated groups (p<0.01). There is no significant differences between the hypoxic group and ischemic+hypoxic group in either serum CK or LDH levels (p>0.05) (Table 4).

**Table 4:** Serum CK and LDH level measuring results in 7-day old rats.

groups	CK (U/L)	LDH (U/L)
The controls	2265±162	1568±66
The hypoxic group	4988±459^**^	2925±255^**^
The ischemic+hypoxic group	5314±437^**^	3133±312^**^

^**^ p<0.01 VS controls

### Elevated inflammatory mediators TNF-α, IL-1B and MMP-9 in the hypoxic treated brain

The levels of TNF-α, IL-1B and MMP-9 in the brain homogenate were higher in the hypoxic group and the ischemic+hypoxic group than the controls (p<0.01 for all three). However, there is no significant differences between the hypoxic and the ischemic+hypoxic group in the level of any of the three inflammatory mediators (p>0.05) (Table 5).

**Table 5:** Expression of inflammatory mediators TNF-α, IL-1B and MMP-9 in the brain.

groups	TNF-α (pg/ml)	IL-1β (pg/ml)	MMP-9 (pg/ml)
The controls	3.50±0.51	2.90±0.47	8.89±1.45
The hypoxic group	15.47±1.80^**^	6.76±0.77^**^	17.03±1.69^**^
The ischemic+hypoxic group	16.77±1.89^**^	7.28±0.92^**^	18.54±1.64^**^

^**^ p<0.01 VS controls

### Sequential pathological change of in HE-stained brain tissue

**Figure 1:**
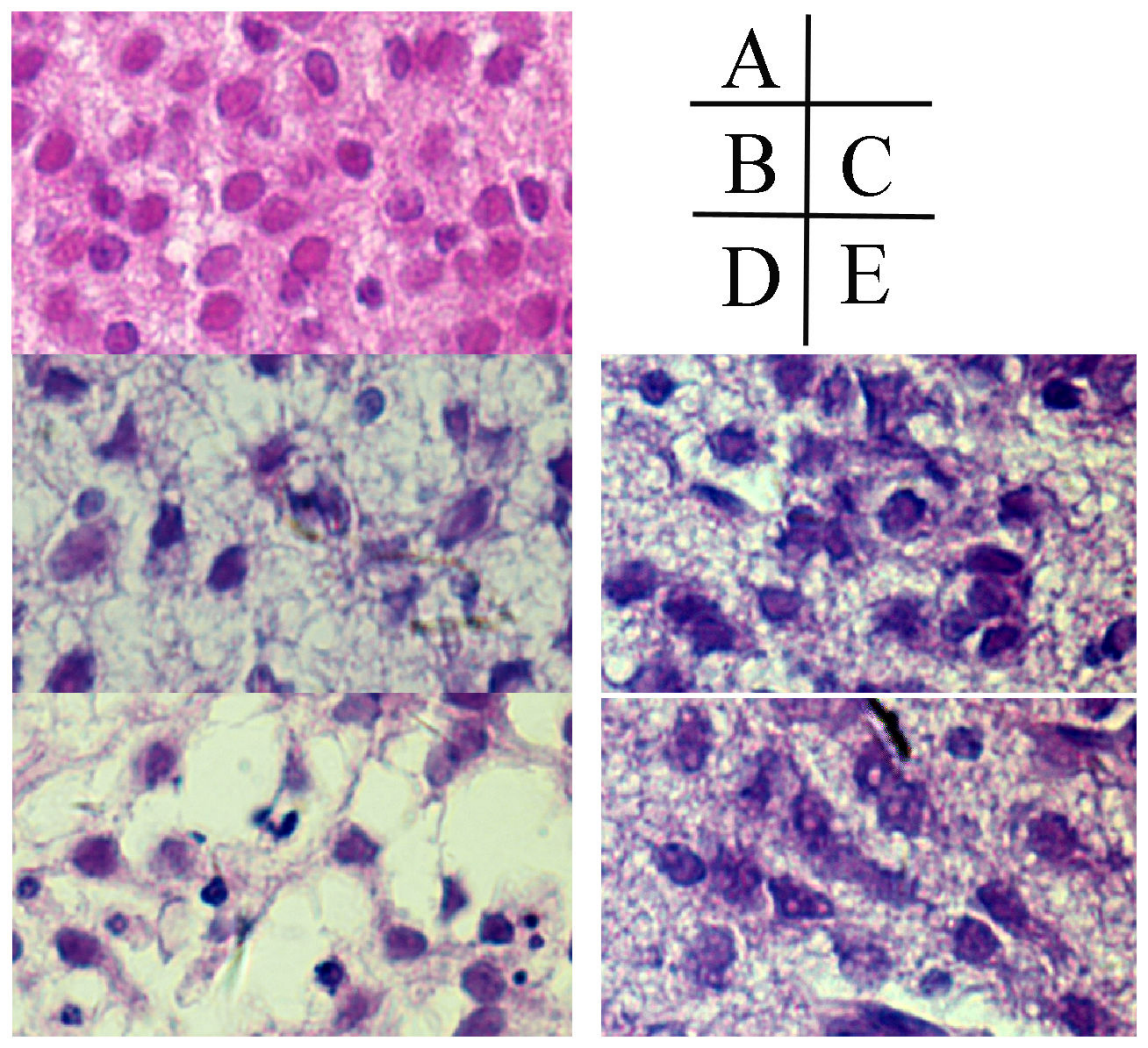
HE staining result of subcortex brain injury tissue (400×) in each group. A Normal control group has normal structure, regular cell arrangement and complete cell outline with intact, center-positioned nucleus and clear entoblast; B, hypoxic 1d; C, hypoxic 3d; D, ischemia+hypoxic 1d; E, ischemia+hypoxic 3d. In the hypoxic group (B to E), we observed leukoaraiosis, abnormal cell arrangement and partial or complete neuron degeneration, at 3d after the experiment, there is apparent recovery in the tissue damages in both the hypoxic group and the ischemic+hypoxic group compared to those observed in 1d. No obviously difference observed in 2 hypoxic treated group.

**Figure 2:**
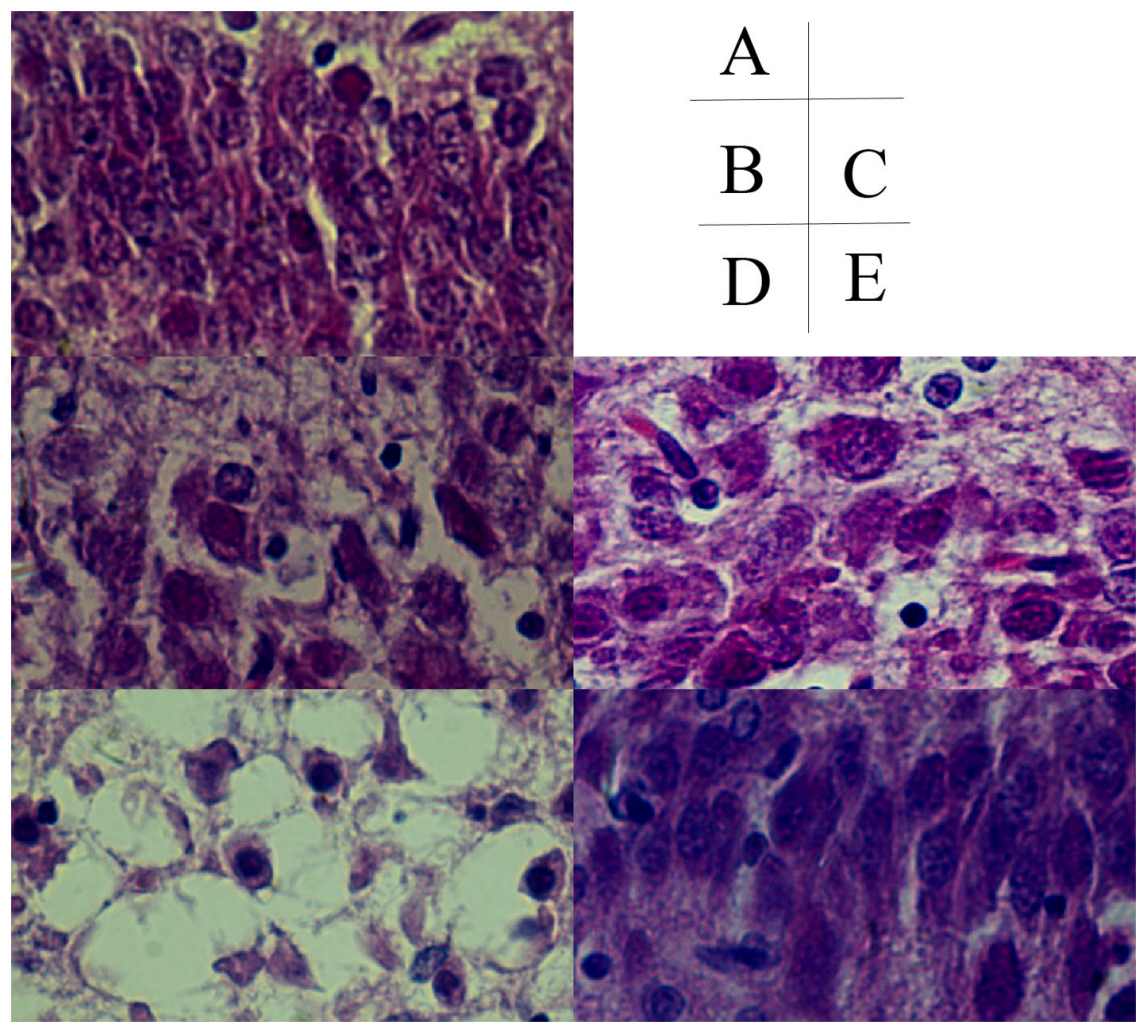
Hippocampus brain injury HE staining result (400×). A, control group; B, hypoxic 1d; C, hypoxic 3d; D, ischemia+hypoxic 1d; E, ischemia+hypoxic 3d.

**Figure 3:**
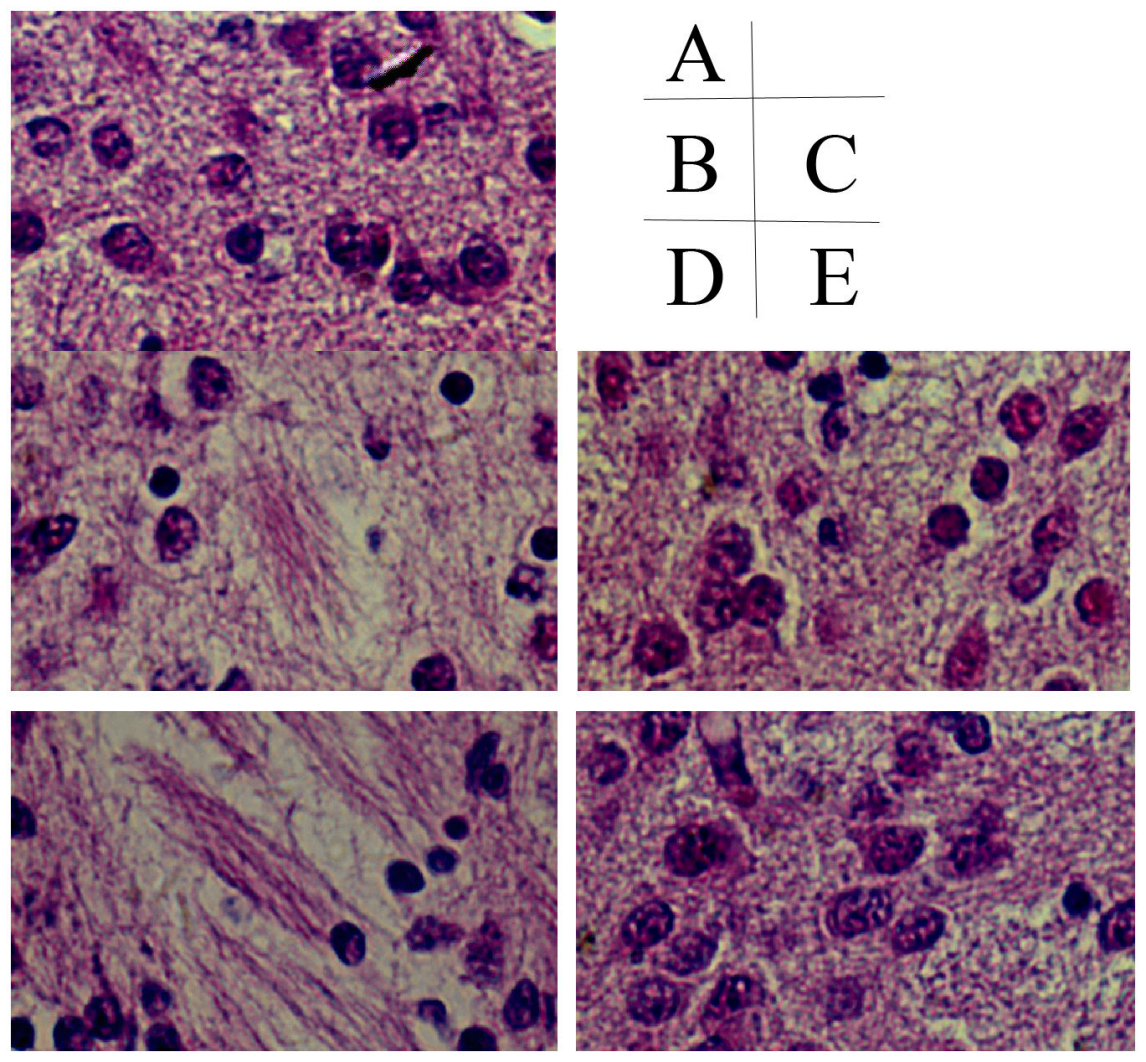
HE staining result of brain injury around lateral ventricle (400×). A, control group; B, hypoxic 1d; C, hypoxic 3d; D, ischemia+hypoxic 1d; E, ischemia+hypoxic 3d.

Figure 1, 2 and 3 were the HE staining results of subcortex brain, hippocampus brain and brain injury around lateral ventricle after 1d and 3d of the experiment. The HE stained brain tissue of the normal control group has normal structure, regular cell arrangement and complete cell outline with intact, center-positioned nucleus and clear entoblast. In the hypoxic group, leukoaraiosis, abnormal cell arrangement and partial or complete neuron degeneration were observed in the cortex and hippocampus after 1d of the experiment. The pathological scores of for these two brain regions in this group were significantly higher than those of the control group (cortex: 19.67±2.52 vs. 3.67±1.15, P<0.01; hippocampus: 12.67±0.64 vs. 2.67±0.58). In the ischemic+hypoxic group, large areas of cell death and integration with nucleus pyknosis and fragmentation, as well as decreased cell density were observed 1d after the experiment. The pathological scores for this group were not only significantly higher than those in the controls (cortex: 39.33±2.51 vs. 3.67±1.15, P<0.01; hippocampus: 41.33±2.08 vs. 2.67±0.58, P<0.01), but also significantly higher than the hypoxic group (cortex: 39.33±2.51 vs. 19.67±2.52, P<0.01; hippocampus: 41.33±2.08 vs. 12.67±0.64 P<0.01). At 3d after the experiment, there is apparent recovery in the tissue damages in both the hypoxic group and the ischemic+hypoxic group compared to those observed in 1d. The pathological scores for both groups were still significantly higher than those in the controls (cortex: 10.66±1.53VS 2.67±0.57, 11.67±1.53 vs. 2.67±0.57, respectively, P<0.01. Hippocampus: 5.33±1.53 vs. 2.33±0.58, 7.00±1.00 vs. 2.33±0.58, P<0.01. However, there is no significant difference in pathological scores between the two experimental groups (cortex, 11.67±1.53 vs. 10.66±1.53, P=0.38; hippocampus, 7.00±1.00 vs. 5.33±1.53, P=0.11). In the area near the lateral ventricle, serious tissue damages were obvious in both the hypoxic group and the ischemic+hypoxic group at 1d (32.00±3.46 vs. 6.00±1.00, 33.00±2.65 vs. 6.00±1.00, respectively, P<0.01). Partial recovery of the damages were also observed in both experimental groups at 3d but the pathological scores were still significantly higher than the control group (21.00±2.36 vs. 4.33±0.58, 23.67±1.03 vs. 4.33±0.58, respectively, P<0.01. There is no significant difference between these two groups at either d1 (32.00±3.46 vs. 33.00±2.65, P=0.21) or d3 (21.00±2.36 vs. 23.67±1.03, P=0.27). The results were shown in Tables 6-8.

**Table 6:** The brain injury pathological score of neonate rat cortex (point).

groups	Pathological score	
	1d	3d
The normal control	3.67±1.15	2.67±0.57
The hypoxic group	19.67±2.52^**^	10.66±1.53^**^
The ischemic+hypoxic group	39.33±2.51^**#^	11.67±1.53^**^
F	205.17	43.80
P	<0.01	<0.01

^**^ p<0.01 VS the normal control, # p<0.01, VS the group anoxic for 1d.

**Table 7:** The brain injury pathological score of neonate rat hippocampus (point).

groups	Pathological score	
	1d	3d
The normal control	2.67±0.58	2.33±0.58
The hypoxic group	12.67±0.64^*^	5.33±1.53^*^
The ischemic+hypoxic group	41.33±2.08^*#^	7.00±1.00^*^
F	725.07	13.73
P	<0.01	0.006

^*^, p<0.05 VS the normal control; ^#^, p<0.01 VS the group anoxic for 1d.

**Table 8:** The pathological score of brain injury beside neonate rat lateral ventricle (point).

groups	Pathological score	
	1d	3d
The normal control	6.00±1.00	4.33±0.58
The hypoxic group	32.00±3.46^**^	21.00±2.36^**^
The ischemic+hypoxic group	33.00±2.65^**^	23.67±1.03^**^
F	190.05	148.20
P	<0.01	<0.01

^**^ p<0.01 VS the normal control.

**Figure 4:**
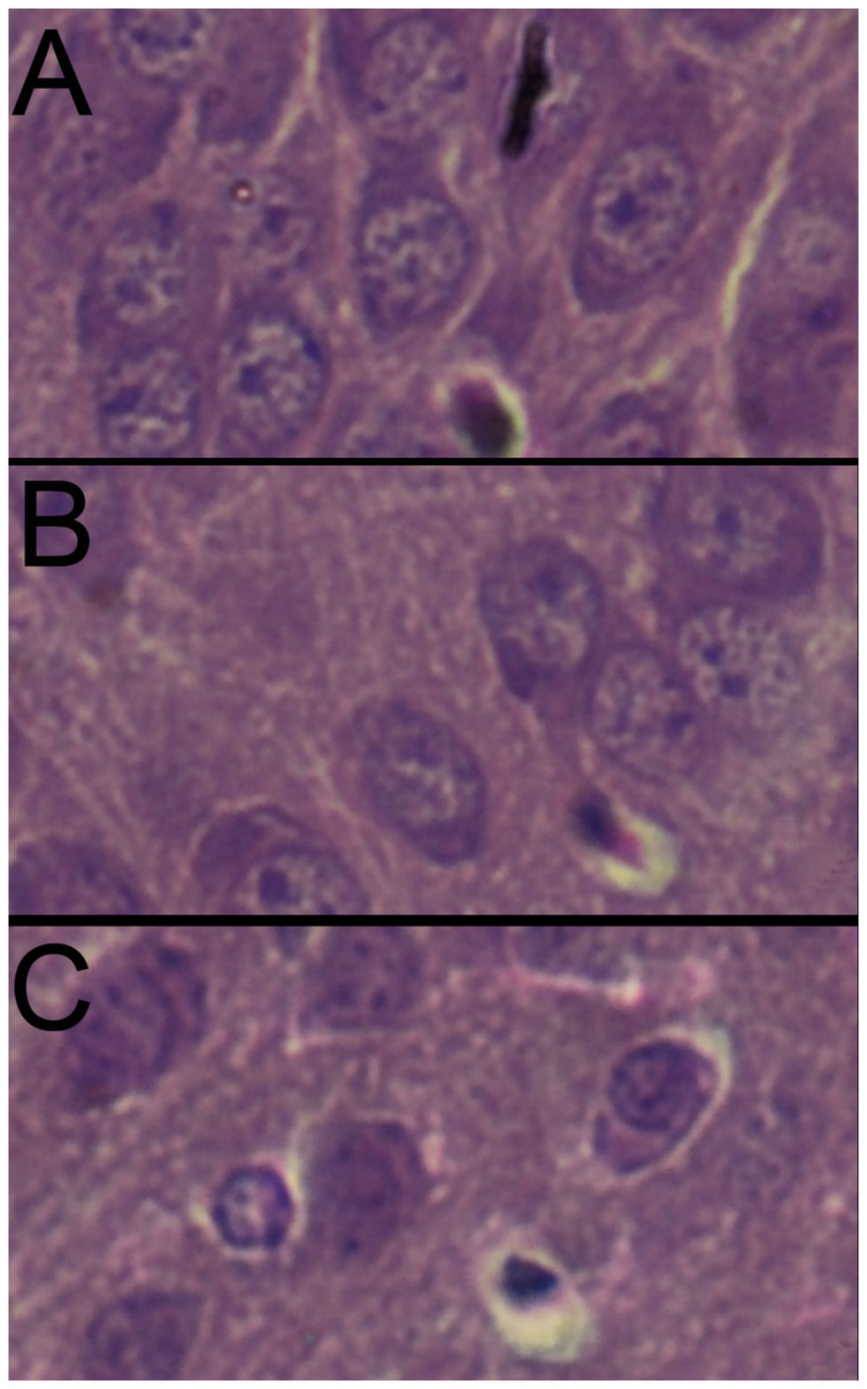
The HE staining result of injury of cortex in 21d neonate rat (400×). A, control group; B, hypoxic group; C, ischemia+hypoxic group.

**Figure 5:**
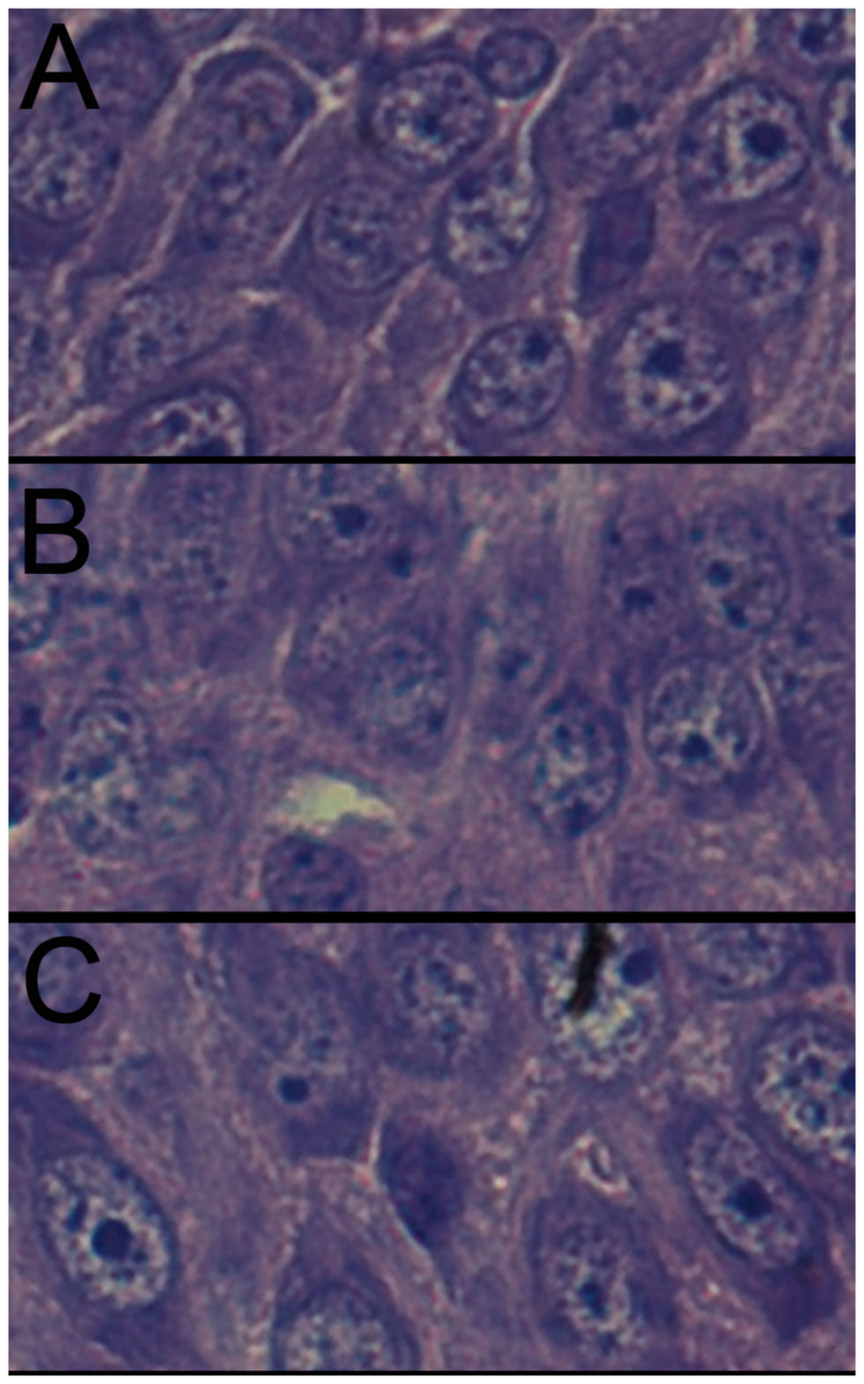
The HE stains result of injury of hippocampus in 21d neonate rat (400×). A, control group; B, hypoxic group; C, ischemia+hypoxic group.

The 21d pathological scores were showed in Table 9, these long term observation showed the recovery of the damages sustained and injury of brain tissue further reduced, only single neuron necrosis could be observed in cortex (Figure 4) and hippocampus (Figure 5), no sheets and focal necrosis be observed. There is statistical difference (P<0.05) between the controls and 2 hypoxic groups, while there is no significant difference between the hypoxic and the ischemic+hypoxic group (P=0.042).

**Table 9:** The 21d- pathological score of cortex and hippocampus in neonate rat (point).

groups	Pathological score	
	cortex	hippocampus
The normal control	2.26±0.43	1.96±0.35
The hypoxic group	8.85±0.89^*^	3.91±1.25^*^
The ischemic+hypoxic group	9.33±1.04^*^	4.14±1.17^*^

^*^ p<0.05 VS the normal control.

### Aggravated *In situ* apoptosis in hypoxic and ischemic+hypoxic group

**Figure 6:**
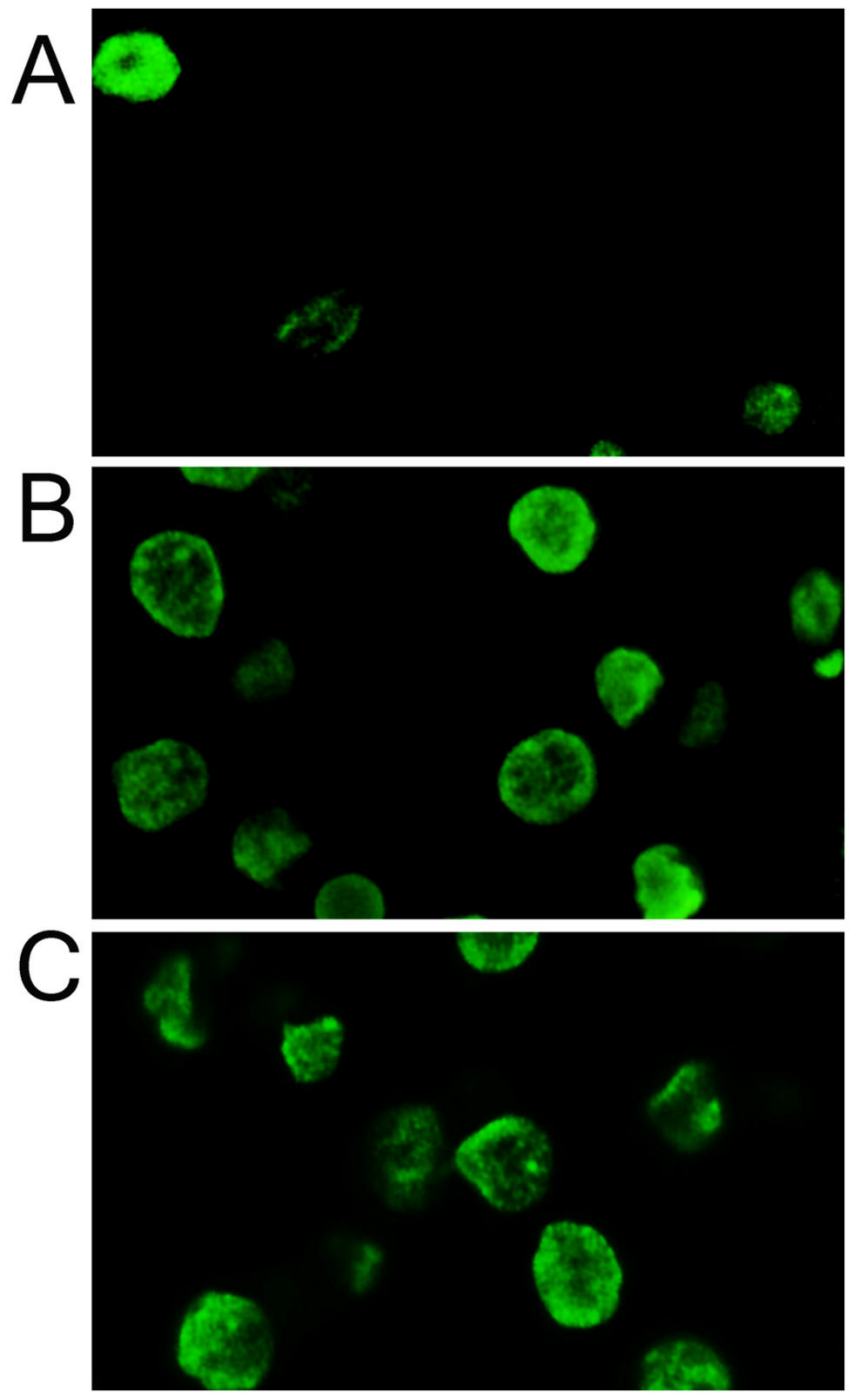
Neural cell apoptosis testing by TUNEL, much more TUNEL positive cells were observed in hypoxic and ischemic+hypoxic group than the controls (400×). A, control group; B, hypoxic 1d; C, ischemia+hypoxic 1d.

As shown in Figure 6, much more TUNEL positive cells were observed in hypoxic and ischemic+hypoxic group than the controls. 

### Expression of c-fos and p-ERK in the brain

**Figure 7:**
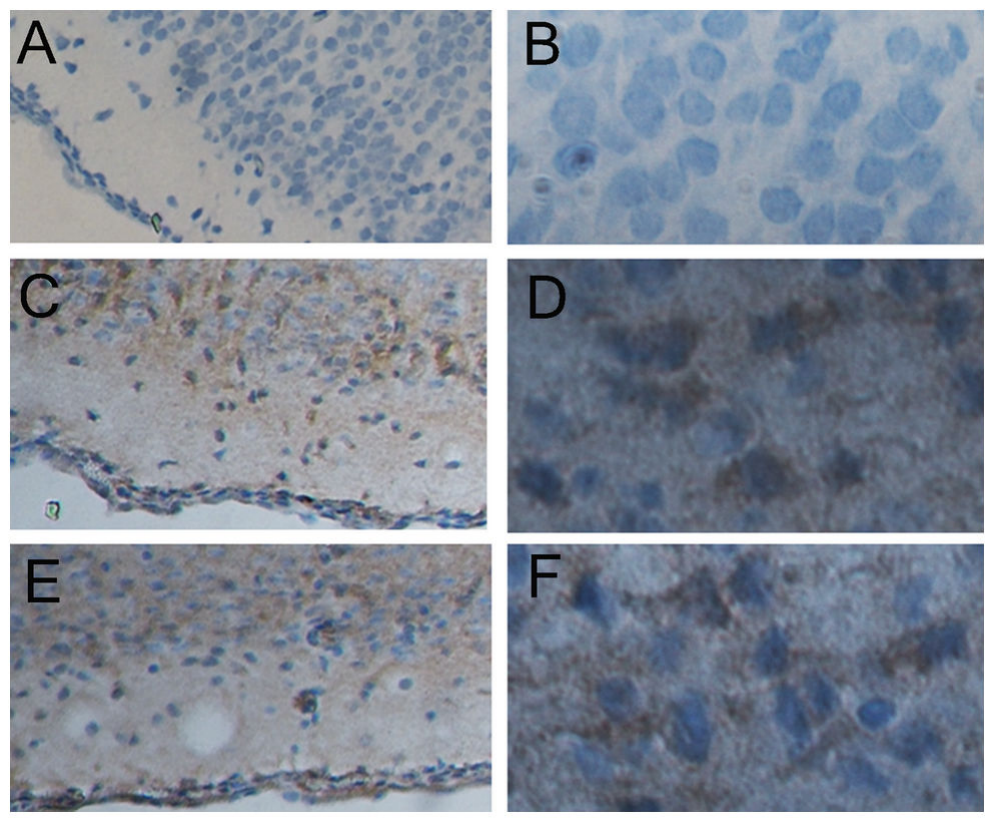
Immunohistochemistry analysis of c-fos expression in the brain of the rats. A, control (100×); B, control (400×); C, hypoxic 1d (100×); D, hypoxic 1d (400×); E, ischemia+hypoxic 1d (100×); F, ischemia+hypoxic 1d (400×).

Immunohistochemistry analysis did not detect c-fos expression in the brain of the control rats. However, c-fos was clearly present in the brain tissues from both the hypoxic group and the ischemic+hypoxic group (Figures 7).

**Figure 8:**
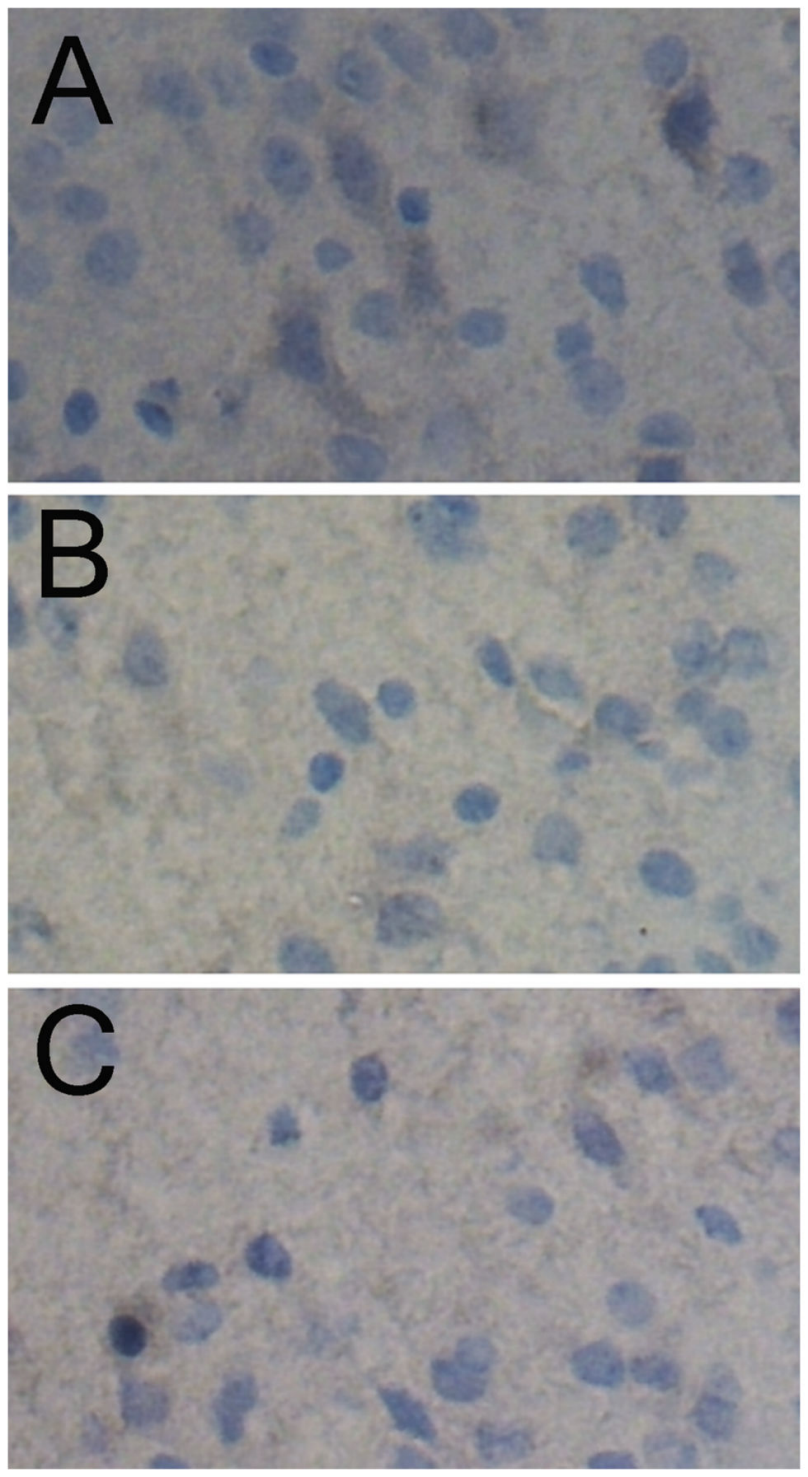
The p-ERK levels in the brains tissues from the hypoxic group and the ischemic+hypoxic group were both lower than that in the brains from the controls by immunohistochemistry analysis. A, control group (400×); B, hypoxic 1d (400×); C, ischemia+hypoxic 1d (400×).

The p-ERK levels in the brains tissues from the hypoxic group and the ischemic+hypoxic group were both lower than that in the brains from the controls (Figure 8).

## Discussion

The HIBD caused by perinatal asphyxia is one of the most devastating diseases commonly seen in neonates. It has a high mortality rate and those who survive may still suffer from developmental disorders in the nervous system. Even with well improved nursing skills and advanced monitoring technologies, there still lacks an efficient detection method and an effective treatment of the disease. It is difficult to develop a treatment for a disease without knowing its pathogenesis [2,12-14]. And a good animal model is essential for researchers to understand the detailed mechanisms of HIBD.

Presently, anoxia during perinatal asphyxia is believed to be the major cause of HIBD and is the focus of many studies on the disease [15]. The most common way of creating HIBD condition in laboratory animals such as neonatal rats is by carotid artery ligation to induce ischemia followed by oxygen deprivation [16,17]. However, surgical procedure is complicated because of the small and thin arteries in neonatal rats. Also, trauma from the incisions and anesthesia can cause high fatality or produce unexpected complications in these young experimental animals, which may create very different situations from the clinical HIBD resulting from perinatal asphyxia. It has been previous shown in pigs that oxygen deprivation alone is sufficient to produce the symptoms of hypoxic brain injuries similar to those seen in animals undergo the combined procedure of carotid artery ligation and oxygen deprivation, raising the question of whether the highly invasive surgery is necessary.

In our study, although both the hypoxic group and the ischemic+hypoxic group both developed hypoxic brain damage, whether or not the animals receives the ligation surgery did not have significant effects on such results as brain water content or basic neural functions. Histology experiment results showed increasing cell death in the hypoxic group and the ischemic+hypoxic group at d1 after the treatment. While the surgery in the ischemic+hypoxic group resulted in significantly higher pathological scores in the cortex and hippocampus than those from both the controls and the hypoxic groups at d1, there was no significant difference in those scores between the hypoxic group and the ischemic+hypoxic group at d3. And the pathological scores of the two groups at d3 were still significantly higher than those of the controls, indicating persistent brain damages in these animals. Also, in the areas besides the lateral ventricle, there was no significant difference in pathological scores between the two experiment groups. The significantly elevated inflammatory mediators as well as increased CK and LDH levels in the hypoxic group and the ischemic+hypoxic group also confirmed brain damage in these two groups at 1d after the experimental treatment. More importantly, since no significant differences were observed between the hypoxic group and the ischemic+hypoxic group in these, it can be concluded that the hypoxic treatment alone is as effective as the ischemic+hypoxic procedure in creating brain injuries. It is possible that the more severe injuries observed in the in the cortex and hippocampus in ischemic+hypoxic group at d1 may be a result of the trauma from the surgery.

The results from the righting reflex and geotactic reflex tests indicated the primary reflexes in the neonatal rats were suppressed. And the results from the vertical screen test and the grid walk test demonstrated brain hypoxic brain damage during early development can lead to impaired motor coordinating ability in later life stages. Again, there is no significant difference between the hypoxic group and the ischemic+hypoxic group, indicating that hypoxic treatment alone is necessary to create long term neural damage in developing rats.

Previous studies have shown that PI3-K/Akt and ERK1/2 pathway can reduce HIBD after ischemia [18]. Our results showed lower p-ERK expression in the brain in hypoxic group and ischemic+hypoxic groups, indicating no activation of the PI3-K/Akt and ERK1/2 pathway in these animals. 

## Conclusion

Eliminating the carotid artery ligation procedure in the process of developing HIBD neonatal rat model has several advantages: first, it makes the procedure easier less time consuming. It is less invasive and traumatic to the animals therefore reducing the stress and fatalities of the animals as well as avoiding potential post-surgery complications. It also reduces human error by eliminating uncertain factors such as skill levels of the operators. Second, this method focuses solely on one of the key factors in HIBD, which is the reduced oxygen supply to the brain therefore it can imitate clinical HIBD process of newborn better. Addition, without the cost for special surgical tools and anesthesia equipment, this method is more affordable with very high success rates. Therefore we believe it is the superior method for generating neonatal rat model for HIBD studies.
